# Old and New Threats—Trace Metals and Fluoride Contamination in Soils at Defunct Smithy Sites

**DOI:** 10.3390/ijerph16050819

**Published:** 2019-03-06

**Authors:** Michał Kupiec, Paweł Pieńkowski, Beata Bosiacka, Izabela Gutowska, Patrycja Kupnicka, Adam Prokopowicz, Dariusz Chlubek, Irena Baranowska-Bosiacka

**Affiliations:** 1 Institute of Socio-Economic Geography and Spatial Management, University of Szczecin, Mickiewicza St. 18, 70-383 Szczecin, Poland; michal.kupiec@usz.edu.pl; 2 Department of Environmental Protection and Development, West Pomeranian University of Technology, Słowackiego 17 St., 71-434 Szczecin, Poland; pawel.pienkowski@zut.edu.pl; 3 Institute of Marine and Environmental Sciences, University of Szczecin, Wąska 13 St., 71-415 Szczecin, Poland; bebos@univ.szczecin.pl; 4 Department of Biochemistry and Human Nutrition, Pomeranian Medical University in Szczecin, Broniewskiego 24 St., 71-460 Szczecin, Poland; izagut@poczta.onet.pl; 5 Department of Biochemistry and Medical Chemistry, Pomeranian Medical University in Szczecin, Powstańców Wlkp. 72 Str., 70-111 Szczecin, Poland; patrycjakupnicka@o2.pl (P.K.); dchlubek@sci.pam.szczecin.pl (D.C.); 6 Institute of Occupational Medicine and Environmental Health, Kościelna 13 St., 41-200 Sosnowiec, Poland; a.prokopowicz@IMP.sosnowiec.pl

**Keywords:** trace metals, fluorides, urban soil pollution, historical industry

## Abstract

The aim of this study was to investigate soil contamination with trace elements and fluoride at sites in Szczecin (NW Poland) where economic activity was historically associated with the use of trace metals. As the Polish legislation does not recognize the lasting impact of historical pollution on soils, land developers are not obliged to determine soil pollution in the new residential areas, including parks and playgrounds for children. Therefore, in this study, at the locations of defunct metalwork enterprises (smithies, foundries, chemical plants, and small metal production plants), which were closed down after World War II, we determined lead (Pb), chromium (Cr), copper (Cu), zinc (Zn), iron (Fe), manganese (Mn), nickel (Ni), mercury (Hg), cadmium (Cd), and cobalt (Co) levels in the soil. In addition, we also determined fluoride (F) levels due to the contemporary fluoride pollution in the area generated by a large chemical plant with a post-production phosphogypsum waste landfill and a power plant complex. Our results show that soil at the sites of now-defunct smithies can still act as a significant source of trace metals. Pb concentration in the surface (0–20 cm) and subsurface (20–40 cm) layers exceeded concentration thresholds for soils with first-degree pollution. The concentrations of Zn and Cu also exceeded their natural background limits. Furthermore, our research indicates an increased concentration of fluoride in surface layers of the soil; however, not exceeding the fluoride content threshold. These observations have important consequences for public health and safety because, presently, the studied sites function as housing estates and other public facilities. Therefore, contaminated soil at these sites may pose a threat to the health of local residents and should be closely monitored for trace metal contamination levels.

## 1. Introduction

In Poland, soil rehabilitation is mandatory when contamination reaches levels that pose a risk to human health and the environment [1]. However, unfortunately legal regulations that allow such remediation activities to be largely avoided if the soil contamination was created before 1980 also exist [2]. As the Polish legislation does not recognize the lasting impact of historical pollution on soils, land developers are not obliged to determine soil pollution in the areas residential areas, including new parks and playgrounds for children. Thus, any such pollution is generally left unremedied and continues to impact the health of local residents. For this reason, we undertook this study to check whether that historic pollution continues to be an issue for centuries after its initial production and whether the sites of older soil contamination should also be subject to compulsory rehabilitation measures.

Pollution of urban soils with trace metals is a serious problem due to the high toxicity of these substances, their high accumulation tendencies, the non-biodegradability of pollutants, and the large number of sources producing them. Ground soils are generally seen as the main deposit of trace metals and other pollutants released from various industrial sources, coal and fuel combustion, vehicle emissions, and emissions from municipal waste disposal [3,4,5,6,7]. Elevated levels of trace metals in urban soils may adversely affect soil ecosystems, human health, and cause numerous problems in local environments. Therefore, the pollution of urban soils with trace metals is a growing worry among researchers, reporting on trace metal contamination in soils around the world [8,9,10,11,12,13,14,15], showing connections between the level of trace metal pollution in a given area and its industrial history [15,16,17,18], and health risks related to trace metal contamination of soils and dust in the immediate vicinity of urban areas [17,19,20]. According to many of those studies, trace metal pollution in urban soil and dust has become a serious threat due to rapid urbanization and industrialization in recent decades, particularly in countries such as China [15,17] and India [21], and due to illegal waste recycling or illegal gold mining, in Africa [21,22,23,24]. Recently, urban soil has also been used as a diagnostic tool for the assessment of environmental conditions affecting human health [19,25,26,27]. Residents of polluted areas can be exposed to trace metals in many ways through the respiratory system, skin exposure, and by drinking contaminated groundwater [28,29,30,31], all of which may lead to adverse effects on residents’ health [32,33].

Fluorides in the air come from both natural sources and as a result of industrial activity. Gaseous fluorides can travel with the wind for considerable distances, contaminating vast areas. The greatest pollution with anthropogenic fluorides occurs in the vicinity of industrial plants producing fertilizers and phosphogypsum waste landfills [34,35]. Chemical plants producing inter alia phosphate fertilizers can significantly affect the ecosystem because during the production of superphosphate, significant amounts of toxic fluorides, mainly in the form of hydrogen fluoride, are released [34]. Industrial sources of fluoride emissions also include aluminum, glass, and iron production, refineries, brickworks, and cement plants [36]. Additionally, during the production of ceramics and enamel, fluorides such as sodium fluoride (NaF), aluminum fluoride (AlF_3_), and cryolite (Na_3_AlF_6_) are emitted into the atmosphere [37].

Intense industrial development of Szczecin in the late 19th and early 20th century ushered in a period wherein significant quantities of environmentally hazardous substances were deposited in the immediate vicinity of cities. Currently, the locations of enterprises using such substances are well documented and their operations are closely monitored, but the sites of the now defunct pollutant-producing activities have largely been forgotten and their present environmental status remains unknown. Historically, small manufacturers and industrial plants were located within the oldest historical parts of Szczecin, so this is where we expect to find particularly high levels of soil contamination. Indeed, various studies report elevated concentrations of metals in such locations, which are often used as sites for residential buildings and recreational facilities [6,9,38]. Therefore, the aim of this study was to investigate soil contamination with trace metals in places where historical economic activity was associated with the use of trace metals. The locations and production profiles of such establishments were determined on the basis of old city records and address books. Sites were selected for analysis to determine the current level of soil contamination resulting from historic trace metal pollution in the area. Simultaneously, due to the presence of nearby chemical plants, a phosphogypsum waste landfill, and power plant complexes, we examined the concentration of fluoride in the soil.

## 2. Materials and Methods

### 2.1. Study Area

#### 2.1.1. Geological Setting

The oldest part of Szczecin (NW Poland) is located on the Western bank of the Western leg of the Odra (Oder) river in its lower section. The relief of the terrain was formed during the recession of glaciers from the youngest glaciation in the Pomeranian phase. The area of the Odra valley was filled with sandy loam in the Holocene. At present, the surface of the accumulation terrace remains under the cover of organic deposits. The peat layer contains mineral deposits, associated with periodic floods and human activity, such as dredging of the riverbed, construction of floodbanks, and embankments for traffic and buildings [39]. The valley of the lower (northern) Odra cuts through the Pleistocene moraine plateau on which the main part of Szczecin is located. This area is dominated by moraine sediments. Strongly eroded patches of tertiary sediments lie beneath the quaternary substrate in the part adjacent to the plateau [40].

#### 2.1.2. Industrial and Settlement History

In the 19th century, Szczecin became the regional center of the metal industry, mainly associated with shipbuilding and machine industries in West Pomerania. Large industrial plants were located along the port harbor, with the function of this area unchanged to this day. The main object of research, however, are the small scattered and numerous production and craft enterprises among the stone residential buildings in the central part of the town. At the end of the 19th century, in the area of dense urban development, small metalworks were mainly located in outbuildings in the backyards [41]. They all ceased to exist during the war, at the latest in 1945, either due to extensive destruction during bombardments. At present, most of these sites continue to function as residential areas. However, these old backyards are changing in function. The buildings are being demolished to give space for recreational areas, lawns, and playgrounds for children.

#### 2.1.3. Industrial Sources of Fluorine

The mass of fluorine of anthropogenic origin, introduced into the environment is estimated in Poland, at about 7000 tones during the year, most of which is a by-product associated with the production of phosphate fertilizer [34]. In the West Pomeranian Voivodeship, the source of fluoride contamination of the Szczecin agglomeration areas are “Police” S.A. Chemical Plant (ZCHP) with the post-production phosphogypsum waste landfill and the “Dolna Odra” Power Plant Complex [42,43,44]. In 1981–1991, the “Police” emitted 12.8–97.0 tons of fluoride annually [44,45]. The phosphogypsum landfill owned by the ZCHP has been in operation since 1969. It covers an area of 270 ha, of which 180 ha are for storage. Due to the amount of waste received (annually about 2.5 million tonnes of phosphogypsum), this landfill is one of the largest industrial storage sites for inorganic chemistry. The air is the main carrier of fluorine, distributing it to all elements of the natural environment where it can accumulate. The 1980s showed that the amount of fluorine compounds emitted by Chemical Plant significantly exceeded the existing standards [44].

### 2.2. Sample Collection

**Figure 1:**
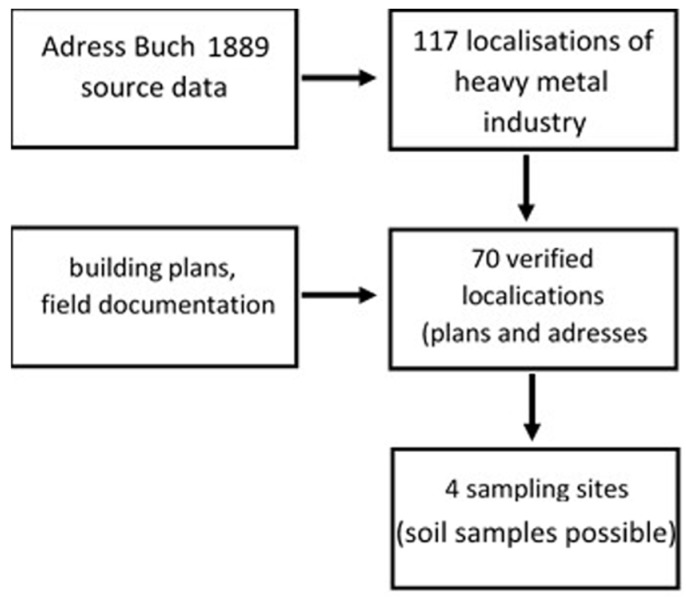
Research preparation. In the first stage of our research, we identified 117 small metalwork enterprises documented in the Stettiner Adress Buch from 1898 and the Building Registration Act of Szczecin. An exact location could be established for 70 plants. As larger areas of exposed soil were rarely available for sampling, 4 optimum sampling locations were selected.

**Figure 2:**
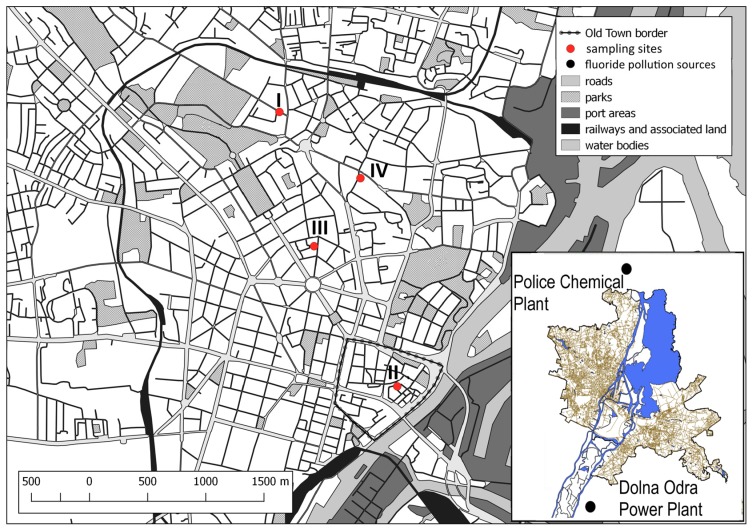
The location of sampling sites in the city of Szczecin (NW Poland). The oldest part of the city. Sampling site I—smithy of horseshoes and vehicle parts; II—smithy of horseshoes and vehicle parts; III—smithy of horseshoes and vehicle parts; and IV—nail smithy.

In the first stage of research, we identified 117 small metalwork enterprises (smithies, foundries, chemical plants, small metal production plants) documented in the Stettiner Adress Buch from 1898 and the Building Registration Act of Szczecin. An exact location could be established for 70 plants (exact location, corresponding to a current addresses). Subsequently, a survey of situational plans was made based on the Archives of the State Building Administration in Szczecin and a field survey of the selected locations, as the larger areas of exposed soil were rarely available for sampling. Consequently, four optimum sampling locations were selected (Figure 1 and Figure 2; Table 1).

The material for testing was collected using a manual drilling device. Samples of soil—20 cm long sections of a 7 cm diameter core (~500 g each)—were taken from a depth of 20–120 cm. At three of the sampling sites 120 cm was the maximum depth, with further drilling impossible due to a concrete slab or brick wall. A sample from a depth of 120–140 cm was only taken in Śląska street.

### 2.3. Sample Characteristics

Material classified as mineral-organic-supplemented anthropogenic rubble ranged from 30% to 70% of the total volume of the collected material. The soil skeleton constituted ~30% of samples, with humus constituting only a small proportion (~5%). The samples were collected with plastic blades in order to avoid metal contamination of samples. 

### 2.4. Quantification of Trace Metals

Soil samples, air-dried and sifted, were subjected to digestion according to EPA method 3050b. Approximately 0.5 g of each soil sample was mixed with 5 mL HNO_3_ and heated at 95 °C for 15 min. Subsequently, 2.5 mL concentrated HNO_3_ (Baker Instra-Analyzed, VWR International, Gdansk, Poland) was added and the digestion was continued for another 30 min. The digestion with concentrated HNO_3_ was repeated until no brown fumes were produced by the sample, indicating complete oxidation by HNO_3_. Next, the sample was evaporated and 2 mL deionized water (MilliQ, Merck, Warsaw, Poland) and 2 mL 30% hydrogen peroxide (Baker Instra-Analyzed) were added. The sample was then partially evaporated and, after the addition of 2.5 mL concentrated HClO_4_, heated for 15 min. After cooling, the resulting solution was quantitatively transferred to a 50 mL volumetric flask and diluted with deionized water to the full volume [46].

Pb, Cr, Cu, Zn, Fe, Mn, and Ni were determined by flame atomic absorption spectrometry (FAAS) in an air-acetylene flame at the most sensitive wavelengths and with optimized parameters using UNICAM 939 Solaar spectrometer (Labexchange Burlandingen, Germany). Hg was determined by cold vapor atomic absorption technic (CVAAS) with the amalgamation step. 1–2 mL aliquots of sample solution were introduced into bubbler containing 50 mL 0.5 M sulfuric acid and 5 mL 20% tin chloride dissolved in HClO_4_. The solution in the bubbler was purged by N_2_ and Hg was trapped on the gold wire, which was subsequently heated to release and detect the collected Hg using UNICAM 939 Solaar spectrometer at 253.7 nm wavelength. Cd and Co were determined by graphite furnace atomic absorption spectrometry (GFAAS) at 228.8 nm and 242.5 nm wavelengths, respectively, with a PerkinElmer 4100ZL spectrometer (Krakow, Poland) equipped with a Zeeman background correction according to Chen and Ma [46]. All the target elements had matrix spike recoveries within 91% and 108%. Standard stock solutions of 1000 ppm Pb, Cr, Cu, Zn, Fe, Mn, Ni, Hg, Cd, and Co were obtained from Central Office of Measures (GUM, Warsaw, Poland) and diluted to appropriate working and calibration standard solutions to prepare calibration curves. Quality control assurance was performed by analysis of matrix match calibration standards, and 15% variance from expected metals concentration was used as a threshold for successful quality control. The limits of detection were approximately 6 ppm for Fe and Mn determination, 2 ppm for Pb and Cr, 1 ppm for Co, Ni, Cu, and Zn, and for Hg and Cd determination the detection limit was 0.02 ppm and 0.01 ppm, respectively.

### 2.5. Determination of Fluoride

Soil samples (10 mg) were incubated for 60 min (95 °C, Thermomix, Eppendorf, Warsaw, Poland) supplemented with 1mL 2 N HClO_4_. After cooling, sodium citrate (2.5 mL) and TISAB II (2 mL) were added to the 0.5 mL of sample. Soil concentrations of F were determined using a potentiometric ion-selective Orion electrode (Thermo Scientific, Warsaw, Poland) according to the work of Gutowska et al. [47]. The fluoride content in samples was calculated based on the difference of potentials measured in each sample, the sample weight and the concentration of the added standard.

### 2.6. Statistical Analysis

The obtained results were analyzed using the Statistica 13.0 software package (Krakow, Poland). The arithmetical mean and SD were calculated for each of the studied parameters. The distribution of results for individual variables was obtained with the Shapiro-Wilk W test. As most of the distributions deviated from the normal distribution, non-parametric tests were used for further analyses. Mann-Whitney U and Wilcoxon signed-rank tests were used to determine the significance of differences between the sampling sites and depths of measurement. The Spearman’s rank correlation coefficient was used to determine the strength of correlations between the parameters. A probability with *p* ≤ 0.05 was considered statistically significant.

## 3. Results

The studies on soil pollution by trace metals in historic small metal plants showed that the mean concentration of all analyzed metals in the soil was 1040.90 mg/kg dry mass (d.m.). The highest mean concentration (1824.56 mg/kg d.m.) was found in the samples taken from a depth of 120–140 cm. The concentration of fluoride in the soil was highest in the upper layers of soil (88.93 mg/kg d.m.) at a depth of 0–20 cm.

### 3.1. Cadmium

**Figure 3:**
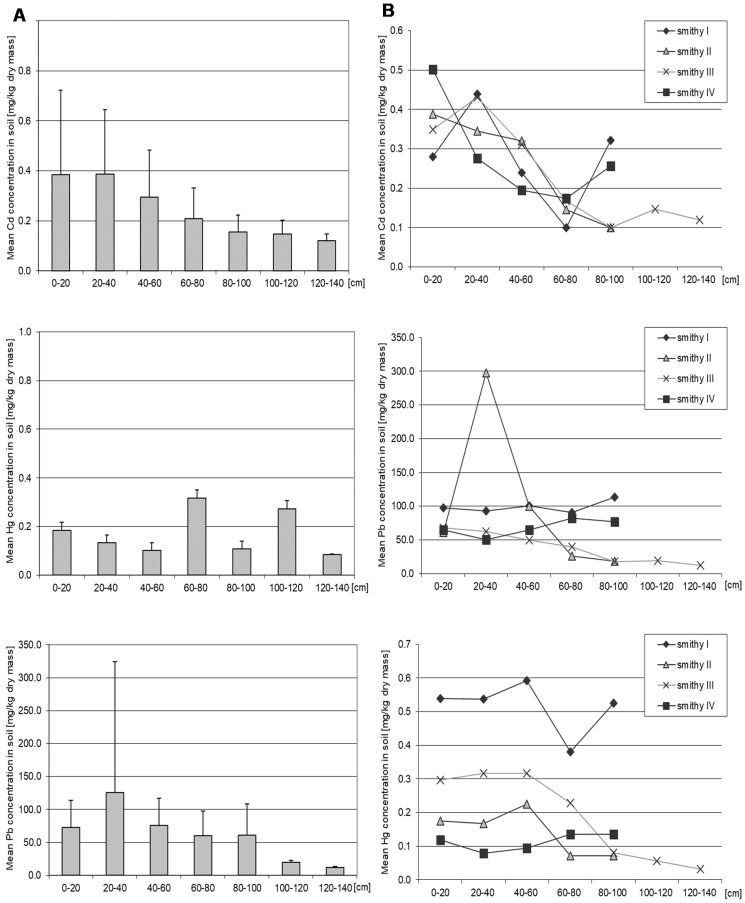
Mean soil Cd, Pb, and Hg levels depending on the depth of sampling (**A**) and depending on the site and the depth of sampling (**B**). Data represent the means ± SD for 3 independent measurements. There is no statistically significant differences in Cd, Pb, and Hg concentration depending on the depth of sampling (Mann-Whitney U test). Statistically significant differences depending on the site of sampling (Wilcoxon test) see Supplementary Table S2.

The mean soil Cd concentration for all sampling sites was 0.28 mg/kg d.m. Cd concentrations in the shallower soil layers was higher than in deeper layers, although the differences were not statistically significant (Figure 3, Table S1). The highest concentration of Cd (1.39 mg/kg d.m.) was obtained at 0–20 cm at the sampling site IV. At two sampling sites (III and IV) mean Cd concentration in the shallow layers (0–20 cm and 20–40 cm) were significantly higher than in the deeper layers of soil (Table S3). Mean Cd concentration also varied depending on the sampling site (Table S2). Differences between sites were observed at 0–20 cm (I vs. III), at 60–80 cm (I vs. III, I vs. IV), and at 80–100 cm between sites III and IV, and between sites II and IV (*p* = 0.04). Mean Cd concentration negatively correlated with the depth of sampling (Rs = −0.51, *p* = 0.05), (Table 2).

### 3.2. Lead

**Figure 4:**
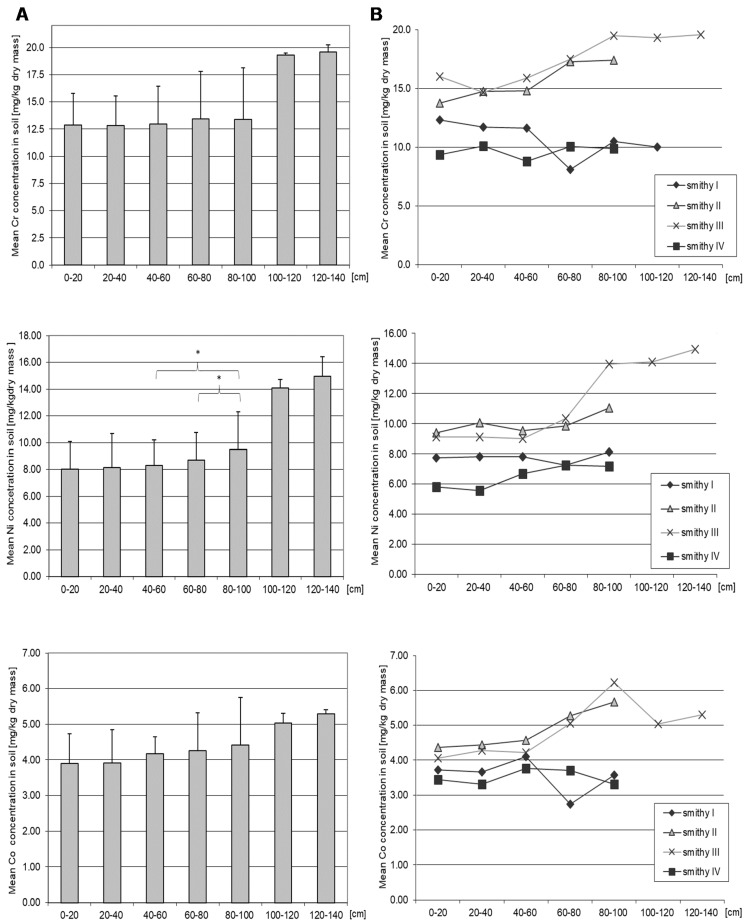
Mean concentration of Cr, Ni, Co in soil depending on the depth of sampling (**A**) and depending on the site and the depth of sampling (**B**). Data represent the means ± SD for 3 independent measurements. There is no statistically significant differences in Cr and Co concentration depending on the depth of sampling (Mann-Whitney U test). * *p* < 0.05, statistically significant differences in Ni concentration depending on the depth of sampling (Mann-Whitney U test). Statistically significant differences depending on the site of sampling (Wilcoxon test), see Supplementary Table S2.

The mean Pb concentration for all studied locations was 80 mg/kg d.m. The highest mean soil Pb was recorded at 20–40 cm (Figure 4, Table S1). At II sampling site it reached 297.62 mg/kg d.m. Below 100 cm, Pb levels were lower than at the surface layers. The values obtained in our research are higher than the average concentration of this metal for the entire city, i.e., 31 mg/kg d.m. [48]. The mean Pb concentration varied significantly depending on the sampling site (Table S2). In the deeper layers (40–100 cm) statistically significant differences were observed between all smithies (*p* = 0.05), but at 0–20 cm and 20–40 cm there were no significant differences. Analysis of Pb concentration at different depths within one site showed statistically significant differences (Table S3). The 0–20 cm and 20–40 cm layers differed in Pb levels from other soil layers at three sampling sites (II, III, IV), while at 60–80 cm, the Pb level differed from deeper soil layers at two sampling sites (II, IV) (*p* = 0.04). At the site II, Pb level at 20–40 cm was higher than at the other layers, while at IV site Pb in that layer was lower than in the other layers. Smithy III had the highest Pb concentration in the surface layer. Lead level at 40–60 cm was higher than at 60–80 cm only at one sampling site (III) (*p* = 0.04). There was a significant negative correlation between the Pb content in the soil and the sampling depth (Rs = −0.31, *p* = 0.03), (Table 2).

### 3.3. Mercury

Mean Hg concentration for the entire soil profile for all sites was 0.19 mg/kg d.m. The highest mean Hg was found at 60–80 cm (0.19 mg/kg d.m.), the lowest at 120–140 cm (0.09 mg/kg d.m.), but the difference between these two values was not statistically significant (Figure 4, Table 2 and Table S1). Mean soil Hg correlated negatively with depth (Rs = −0.29, *p* = 0.05). Hg levels depended significantly on the sampling site (Table S2). At site I Hg concentration was the highest in the entire soil profile and was significantly higher than at sites IV (*p* = 0.004) and III (*p* = 0.03), and II (*p* = 0.03). The differences were also observed between sites II and III and III and IV. A significant correlation between Hg concentration and depth of sampling was observed only at the site IV (nail smithy) (*p* = 0.04). The layer 0–20 cm had a higher Hg than at 20–40 cm, and at 20–40 cm Hg level was lower than at the deeper layers (*p* = 0.04) (Table S3).

### 3.4. Chromium

Mean Cr level for the entire soil profile at all four locations was 13.39 mg/kg d.m. The highest mean Cr (19.56 mg/kg dm) was found in the deeper layers (100–140 cm), but not significantly different from the other soil layers (Figure 4, Table 2 and Table S1). There were statistically significant differences in chromium concentration between all sites of soil sampling (Table S2). Differences in mean Cr were observed at soil layers from 0 cm to 100 cm between sites I and II (*p* = 0.04), I and III (*p* = 0.04), III and IV (*p* = 0.004), and II and IV (*p* = 0.004), and sporadically between other site pairs at 0–20 cm, 40–60 cm, and 80–100 cm. Analysis of Cr concentration at different depths within one site showed statistically significant differences in concentration between the surface layer and the deeper layers of soil (*p* = 0.04) (Table S3).

### 3.5. Nickel

The mean concentration of nickel in the entire soil profile at all sites was 8.79 mg/kg d.m. Significant differences in Ni concentrations in soil were observed between 40–60 cm and 80–100 cm (*p* = 0.02) and between 60–80 cm and 80–100 cm (*p* = 0.02) (Figure 4, Table S1). Significant statistical differences in average concentration of Ni in soil were recorded between all sampling points at almost all soil depths (Table S2). Depth-related differences in Ni content were observed for site IV, where the concentrations of Ni in the shallower layers of soil were significantly lower than in the deeper layers (*p* = 0.04), and at site III (where Ni levels were the highest) between layers 20–40 cm and 60–80 cm (*p* = 0.01) and between 40–60 cm and 60–80 cm (*p* = 0.01) (Table S3). There was also a slight negative correlation between concentration and depth of measurement (Rs = 0.28, *p* = 0.05), (Figure 4, Table S1).

### 3.6. Cobalt

The mean concentration of Co in the entire soil profile in all locations was 4.16 mg/kg d.m. Cobalt concentrations in the soil showed significant differences depending on the sampling site (except site II vs. III), most often in the surface layer and at 60–100 cm. In addition, significant differences in the subsurface layer were observed between the nail smithy (site IV) and the smithy of vehicle parts (site II) (*p* = 0.01) (Table S2). Co concentrations showed significant depth-related differences at two locations: At the nail smithy (site IV) between 20–40 cm and 60–80 cm and between 60–80 cm and 80–100 cm (*p* = 0.01), and at site III between the soil layers at 0–20 cm and 60–80 cm, 20–40 cm and 60–80 cm and between 40–60 cm and 60–80 cm (*p* = 0.01) (Table S3). A positive correlation was observed between the depth of measurement and the concentration of cobalt in the soil (Rs = 0.25, *p* = 0.05).

### 3.7. Manganese

**Figure 5:**
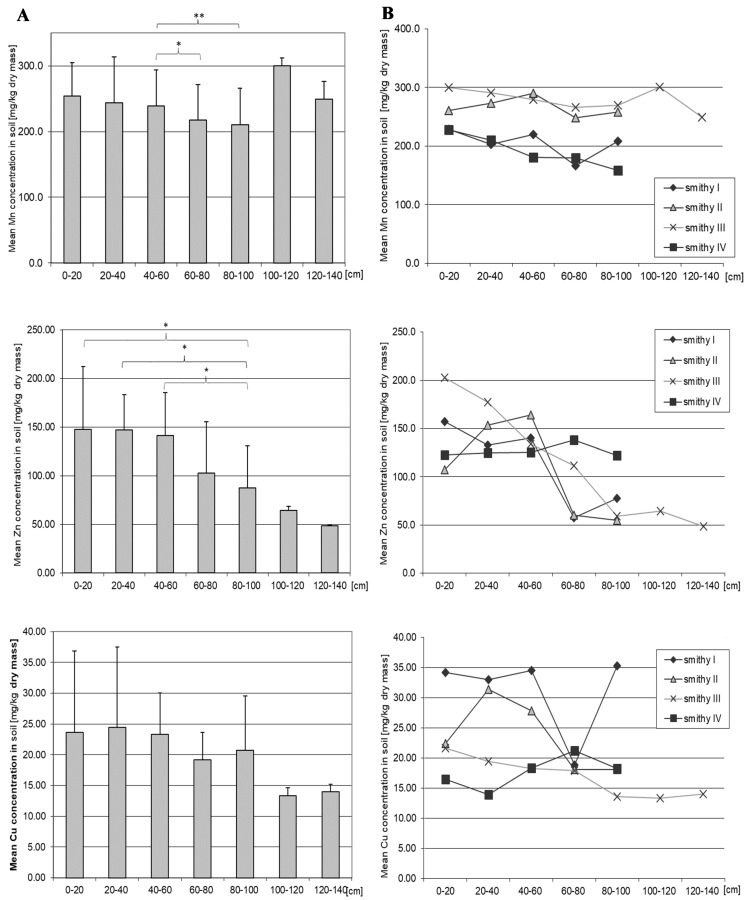
Mean concentrations of Mn, Zn, Cu in the soil depending on the depth of sampling (**A**) and depending on the site and the depth of sampling (**B**). Data represent the means ± SD for 3 independent measurements. * *p* < 0.05, ** *p* < 0.005 statistically significant differences in Mn and Zn concentration depending on the depth of sampling (Mann-Whitney U test). Statistically significant differences depending on the site of sampling (Wilcoxon test), see Supplementary Table S2.

The mean concentration of Mn measured at all locations throughout the depth of the soil profile was 238.09 mg/kg d.m. The mean highest Mn concentration was found at a depth of 100–120 cm, with the lowest at a depth of 80–100 cm, although this difference was not statistically significant (Figure 5, Table S1). Significant differences in the concentration of this element in the soil were observed between the depths 40–60 cm vs. 60–80 cm (*p* = 0.05) and 40–60 cm vs. 80–100 cm (*p* = 0.005). There were significant differences in the concentration of this element depending on the sampling site and depth (Tables S2 and S3). Significant differences between sampling sites at 0–20 cm and 20–40 cm soil layers were only observed between smithies III and IV, and between sites I and III (*p* = 0.01). More frequent were differences at deeper soil layers, observed between almost all smithies (except between smithies II and III) (*p* = 0.05). At two sampling sites (III, IV), Mn concentrations at 20–40 cm were significantly lower than at deeper soil layers (*p* = 0.04). At site I, it was also significantly higher in the 0–20 cm layer than at 20–40 cm (*p* = 0.01).

### 3.8. Zinc

The mean Zn concentration in the soil at the four sites was 125.23 mg/kg d.m. It also significantly differed from the average concentration of this element in the soils of the entire city (22 mg/kg d.m.) and the country (32.40 mg/kg d.m.) [35]. Mean Zn concentration in the shallow soil layers was significantly higher than in the deeper layer of 80–100 (*p* = 0.03). There were significant differences in the concentration of this element depending on the site and depth of sampling (Tables S2 and S3). The site IV had a significantly higher Zn level at 80–100 cm than at sites II and III (*p* = 0.004) and at 60–80 cm than at sites I and II (*p* = 0.004). Zinc levels at sites I and III were significantly higher than at site II (*p* = 0.03), and at site III it was lower than at site I (*p* = 0.03). At surface and subsurface layers (i.e., from 0 cm to 40 cm) differences in Zn occurred only sporadically (III vs. IV, II vs. III) (*p* = 0.01). Zn concentration correlated negatively with the measurement depth (Rs = −0.49, *p* = 0.05).

### 3.9. Copper

The mean concentration of copper in the whole soil profile at the four sites was 22.07 mg/kg d.m. There were significant differences in Cu concentration depending on the site and the depth of sampling (Supplementary Tables S2 and S3). It differed statistically significantly at 0–20 cm (II vs. IV, III vs. IV) (*p* = 0.002) and at 20–40 cm between sites II and III and between sites II and IV (*p* = 0.0002). Differences were also observed at lower soil layers (II vs. III, II vs. IV and I vs. I, II, IV) (*p* = 0.03). Depending on the depth, at three sites (II, III, IV) we found differences between the layer 20–40 cm and other layers (*p* = 0.04), and also between the layer of 0–20 cm and deeper layers (*p* = 0.04). Copper concentrations were lower at site IV (former nail smithy), the areas occupied by smithies for vehicle parts exhibited higher Cu levels.

### 3.10. Iron

**Figure 6:**
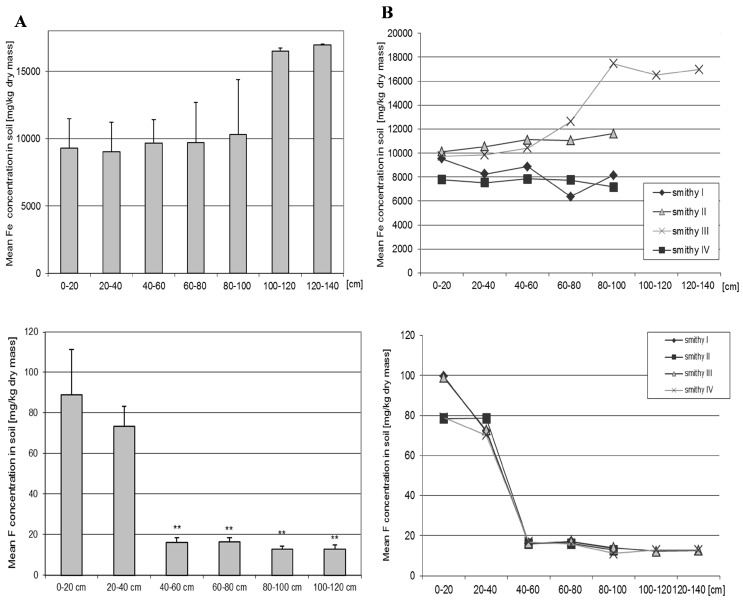
Mean concentration of Fe and F in the soil depending on the depth of sampling (**A**) and depending on the site and the depth of sampling (**B**). Data represent the means ± SD for 3 independent measurements. There is no statistically significant differences in Fe concentration depending on the depth of sampling (Mann-Whitney U test). **p < 0.005 for the significance of difference marked depths vs 0-20cm and 20–40cm. Statistically significant differences depending on the site of sampling (Wilcoxon test), see Supplementary Table S2.

The mean concentration of Fe in the soil was moderate across the entire soil profile and on average was 9930.90 mg/kg d.m. The highest Fe concentration was obtained in the deep soil layer, but this value was not statistically significantly different from the shallower soil levels (Figure 6, Table S1). There were significant differences in the concentration of Fe depending on the site and depth of sampling (Tables S2 and S3). The greatest number of differences were found between sites II and IV, and between sites III and IV (*p* = 0.04). At site IV, where the Fe concentration was the lowest, significant differences in Fe concentrations were observed between deeper soil layers (40–100 cm) (*p* = 0.04).

### 3.11. Fluoride

The concentration of fluoride in the soil was the highest in the upper layers of soil. At a depth of 0–20 cm it was on average 88.93 mg/kg d.m. The concentration was significantly different from the lower layers (40–60 cm) and 60–80 cm (*p* < 0.001); 80–100 cm (*p* < 0.001) and 100–120 cm (*p* < 0.001). F content in layer 20–40 cm was also high and amounted to 73.32 mg/kg dm. It did not differ significantly from the content of F in the surface layer, however, it was statistically significantly higher than the content in deeper layers 40–60 cm (*p* = 0.001), 60–80 cm (*p* < 0.001), 80–100 cm (*p* < 0.001), and 100–120 cm (*p* < 0.001). Similar differences were found depending on the depth of sampling among each site. No significant differences were found in the concentration of F depending on the sampling site.

## 4. Discussion

Analysis of the concentrations of trace metals and fluorides in soils at sites of current and historical industrial activity can be important for preserving the quality of life and health of residents in such areas. Additionally, such research helps guide the choice of measures used to reduce pollution levels and improve urban ecosystems. In Poland, soil rehabilitation is mandatory when contamination reaches levels that pose a risk to human health and the environment [1]. However, unfortunately legal regulations that allow such remediation activities to be largely avoided if the soil contamination was created before 1980 also exist [2]. Thus, pollution such as that identified in this study is generally left unremedied and continues to impact the health of local residents. For this reason, we undertook this study to demonstrate that historic pollution continues to be an issue for centuries after its initial production and that sites of older soil contamination should also be subject to compulsory rehabilitation measures.

This study presents important information about present soil contamination in areas of the city that were the historic locations of smithies. We found higher concentrations of Pb, Zn, Ni and Cu at these sites compared to the average concentrations of these elements in the soils of the entire city agglomeration [48,49,50,51]. The mean soil Cd concentration for all sampling sites in our study was lower than the permissible Cd value for soils with the first-degree pollution, as defined by the Institute of Soil Science and Plant Cultivation (IUNG) in 2017—0.5 mg/kg d.m. [49], however, its concentration was higher than the mean Cd concentration in Polish soils (0.21 mg/kg d.m. [35]). The accumulation of metals in soils collected from the locations of old smithies was also higher than in soil samples from the urban areas of some other world cities with rich industrial pasts, such as Hong Kong (China), Nanjing (China), Sevilla (Spain), and Warsaw (Poland) [8,52,53], Table 3. Our research suggests a link between the activity of late 19th and early 20th century smithies and current trace metal pollution of local soils. These data are in agreement with previous reports in which current trace metal soil contamination is attributed to industrial activity at certain sites [15,16,17,18] including historic metal processing plants [54]. Other studies also show the accumulation of heavy metals in urban soils, e.g., in Cracow (Poland), where metal processing plants have been operating as early as the Middle Ages [55,56].

A comparison of our results with the standards presented in the Ordinance of the Polish Minister of Environment and IUNG 2017 guidelines allowed us to assess the degree of trace metal contamination at the studied sites [49,50]. We also took into account the classification of soil contamination by Kabata-Pendias [35] (Table 3). Based on these guidelines, we identified that at our study sites, soils were characterized by either 0, 1st, and 2nd degree pollution (for Pb, Zn, and Cu) [49,50]. This concern is supported by previous research [19,25,26,27], indicating that soil can act as a potential source of exposure to trace metals leading to adverse effects on human health. The accumulation of toxic elements in surface and subsurface soil layers is closely related to anthropogenic pollution [61]. Zn and Pb in soil are often derived from the same sources of industrial pollution. Glennon et al. associate high soil Zn levels with proximity to industrial plants. Zn, and similarly Pb, is emitted into the atmosphere by metallurgical foundries and workshops and then accumulates in surrounding soils [54]. In our study, the mean concentration of Pb at historic smithy sites was significantly higher than the average for the entire city (31 mg/kg) [50], demonstrating the negative environmental impact of these enterprises. These results show that various elements, including toxic trace metals, remain in soil at the sites of historic smithies for many years after operations have ceased. While few comprehensive city-wide studies of soil Pb contamination have been conducted, the few existing reports indicate that high soil Pb levels can be found in older housing communities, heavily trafficked roads, and around point source Pb emissions stacks [62,63,64]. A reservoir of highly bio-accessible Pb in urban soil and dust derived from that soil is concentrated near the surface, where it is available to be resuspended in the air during dry periods [64,65,66,67]. Polluted soil is a well-known source of human exposure to trace metals, including Pb [57,68]. For example, high serum Pb levels are reported in children living in areas with rich industrial histories [69]. Another study shows that blood Pb levels in two-year-old children correlate with its levels in local dust and soils [69,70,71]. Similarly, people living in areas with soil Cu levels exceeding 80 mg/kg d.m. display an increased number of white blood cells, platelets, increased erythrocyte sedimentation rate, and increased DAS28—factors that aggravate the symptoms of rheumatoid arthritis [72]. Significantly, in our study, soil Cu reached as high as 2nd degree contamination at some studies points. The bioavailability of metals in soil depends on many factors, including its acidification, hydration, content of organic and inorganic matter, and texture [56,73,74], which influence the inclusion of such metals in the food chain [38,75]. In our study, soils from sites in the oldest part of the city contained higher levels of trace metals than soils from forests surrounding the city and garden plots in the city [70].

Our research showed high accumulation of some elements in the studied soils (Cd, Hg, Ni, Pb, and Zn). This observation is significant because of the strong carcinogenic effects of Cd [75], neurotoxic effects of Hg and Pb [75,76], the role of impaired Zn homeostasis in neuronal degeneration and Alzheimer’s disease [77], and the strong allergenic action of Ni [78]. The increased concentrations of these toxic metals found in our study are worrisome due to the presence of housing estates and other public facilities on the surveyed sites. This significantly increases the likelihood of human exposure to the metals contained in soil and their absorption via air and food, which can lead to poisoning and serious detrimental effects on health. It is therefore necessary to conduct further studies on soil contamination in places with high levels of toxic metals to determine their bioavailability and mobility. It is important to note, however, that their elevated concentrations in the studied soil samples are immediately alarming, and the areas where trace metal pollution reaches such high levels should receive swift dedicated treatment. The state of the soil is checked during routine environmental reviews, but unfortunately such examinations are rarely performed in areas with fixed functions, such as housing. In practice, regulations on soil quality standards are more often implemented when developers seek the approval of local environmental authorities for land development projects. In these cases, a thorough analysis of contamination is carried out, including preparation of a report on the environmental impact of the proposed development. Relevant authorities are then able to enforce land rehabilitation measures as compulsory conditions for project approval.

In our research, the concentration of F in surface layers (from 0–40 cm deep) at all examined sites was significantly higher than in deeper soil layers, indicating that fluoride pollution occurred recently. In mineral soils, fluoride content ranges from 0.2–0.3 g/kg, while in soils formed from fluoride-containing minerals, levels range from 7 to 30 g/kg [37]. Increased F concentrations in soil are likely an effect of pollution caused by the operation of chemical plants, both currently and historically. The increase in F concentration in the upper soil layer may be caused by the deposition of fluorine gas compounds originating from the emissions of nearby industrial chemical plants and post-production landfills. A fluoride content of 1 μg/m^3^ in the air causes an annual increase in the content of fluorides in soil by 0.004 to 0.018 g/kg [37,79]. The concentration of fluorides in the soil is also affected by the granularity of its individual fractions, soil pH, and chemical composition (including concentration other elements such as Fe, Si, and Ca) [80]. Fluorine compounds found in soils from the Szczecin agglomeration are consistent with the characteristic pollution found in this area. Calculated distribution, based on data from 1983, showed that, at this time, in the area approximately 8 km from the ZCHP, the instantaneous concentration of fluorine compounds in the air was 877 μg/m^3^, around 30 times higher than the allowable concentration. The mean annual concentration in the air was then 16 μg/m^3^, 10 times higher than permissible. In 1992, as a result of limits on emissions of fluorine compounds, their maximum instantaneous concentration in the Police region was reduced to three times the allowable level and the average annual concentration only twice higher [43]. In recent years, thanks to various eco-investments, emission of fluorine around the ZCHP is stable and lower than the admissible values (2 μg/m^3^) in the air during the year, in accordance with the Regulation of the Minister of Environmental Protection, Natural Resources and Forestry of April 28, 1998, regarding the permissible values of concentrations of pollutants in the air (Journal of Laws of 1998, No. 55, item 355). Similar observations have been made concerning the F content in precipitation. A study investigated the fluorine content in rainfall in the area of impact of the ZCHP from 1977–1996. In the years 1981–1988, fluorine content was twice smaller than the content of fluorine in precipitation recorded in 1978–1980 (it was then an average of 589 kg/km^2^/year), and from 1989 to 1996, it was almost three times smaller, indicating a reduction in fluoride emissions in recent years. As well, the fluorine content in rainfall increased with increasing proximity to the emission source [44]. As a result of the installation of a dual fluorine absorption station in the beginning of 1990s, fluoride pollution of the environment by contaminated rainwater was reduced.

The threat posed by the presence of trace metal and fluoride in soils is still not fully understood [43]. Risk is associated with both the quantity and the mobility of these contaminants in the soil [74]. Not only agricultural soils, but also those intended for housing estates, playgrounds and other public buildings, should be thoroughly inspected to protect the health of local residents.

## 5. Conclusions

Our study suggests a link between the activities of late 19th and early 20th century smithies, currently operating chemical plants, and trace metal and fluoride pollution of the soil. We also showed that the concentration of some of the analyzed metals exceeded their natural background limits, indicating potential risks to human and environmental health. The greatest threat was posed by Pb, Zn, and Cu, whose concentrations were classified as the first and second-degree pollution. Our results indicate that all soils, not only those used for agricultural purposes, should be monitored for trace metal and fluoride contamination.

## Figures and Tables

**Table 1:** Selected research sites.

Facility	Current Function	Location
(I) smithy of horseshoes and vehicle parts	Parking space	Niemierzyńska Str. 3
(II) smithy of horseshoes and vehicle parts	Small park	Grodzka Str. 10
(III) smithy of horseshoes and vehicle parts	Small park	Śląska Str. 29
(IV) Nail smithy	Kindergarten playground	Ofiar Oświęcimia Str. 12

**Table 2:** Concentration of the tested elements (mg/kg d.m.) depending on the depth of sampling and limits and global average concentrations (mg/kg d.m.) of selected elements in the soil. Lead (Pb), chromium (Cr), copper (Cu), zinc (Zn), iron (Fe), manganese (Mn), and nickel (Ni) were determined by flame absorption spectrometry (FAAS) in an acetylene-air flame, while mercury (Hg) was determined by cold vapor using a UNICAM 939 Solaar spectrometer. Cadmium (Cd) and cobalt (Co) were determined by graphite furnace atomic absorption spectroscopy (GFAAS) and a PerkinElmer 4100ZL spectrometer equipped with a Zeeman background correction system.

		Element									
		Cd	Pb	Hg	Cr	Ni	Co	Mn	Zn	Cu	Fe
		**Mean Concentrations (mg/kg d.m.)**									
Depth (cm)	0–20	0.384	72.758	0.185	12.843	8.018	3.898	254.204	147.334	23.683	9297.018
	20–40	0.386	125.632	0.134	12.796	8.142	3.921	244.170	147.016	24.457	9039.099
	40–60	0.295	75.717	0.102	12.939	8.321	4.170	239.315	140.999	23.322	9662.139
	60–80	0.209	60.082	0.318	13.407	8.720	4.252	217.856	102.842	19.213	9691.489
	80–100	0.155	60.844	0.109	13.378	9.500	4.417	210.633	87.265	20.721	10,313.838
	100–120	0.119	19.320	0.273	19.301	14.099	5.040	300.485	64.471	13.355	16,501.389
	120–140	0.120	12.200	0.090	19.560	14.950	5.300	249.480	48.670	14.020	16,969.090
		**Thresholds (mg/kg d.m.)**									
Surface layer of the soil [49]		0.5	50	2	150	25	20	-	70	25	-
Deeper layers of the soil [50]		5	100	3	150	50	30	-	350	100	-
Mean concentration in world soils [35]		1.1	27	0.1	42	18	6.9	418	62	14	3.5% *

* Mean world content (%).

**Table 3:** The average soil metal concentrations (mg/kg d.m.) in urban areas of different world cities.

City	Pb	Zn	Cu	Ni	Cr	References
Warsaw (Poland)	53	140	25	-	13	[53]
Sevilla (Spain)	137	145	68	22	39	[8]
Ghaziabad (India)	112	113	27	-	24	[25]
Hong Kong (China)	95	125	23	12	23	[52]
Beijing (China)	66	87	71	22	-	[57]
Nanjing (China)	104	96	104	-	97	[58]
Changsha (China)	89	276	51.4	-	121	[59]
Shanghai (China)	70	301	59	31	108	[60]

## Supplementary Materials

The following are available online at https://www.mdpi.com/1660-4601/16/5/819/s1, Table S1. Concentration of the tested elements (mg/kg d.m.) in the soil depending on the depth of sampling, Table S2. Statistical significance of the tested elements in the soil depending on the site of sampling, Table S3. Statistical significance of the tested elements in the soil depending on the depth of sampling.
