# Supplementary material for: Old and New Threats—Trace Metals and Fluoride Contamination in Soils at Defunct Smithy Sites
Source: Int J Environ Res Public Health. 2019 Mar 6;16(5):819. doi: 10.3390/ijerph16050819 (PMC6427284; doi:10.3390/ijerph16050819)
Supplement: Supplementary file 1 [file ijerph-16-00819-s001.pdf]

Supplementary Materials:

Table S 1. Concentration of the tested elements (mg/kg d.m.) in the soil depending on the depth of sampling. Lead (Pb), chromium (Cr), copper (Cu), zinc (Zn), iron (Fe), manganese (Mn) and nickel (Ni) were determined by flame absorption spectrometry (FAAS) in an acetylene-air flame, while mercury (Hg) was determined by cold vapor using a UNICAM 939 Solaar spectrometer. Cadmium (Cd) and cobalt (Co) were determined by graphite furnace atomic absorption spectroscopy (GFAAS) and a PerkinElmer 4100ZL spectrometer equipped with a Zeeman background correction system.

	**Depth (cm)**						
	**0-20**	**20-40**	**40-60**	**60-80**	**80-100**	**100-120**	**120-140**
**Cd [mg/kg d.m.]**							
Mean	0.384	0.3856	0.295	0.209	0.155	0.119	0.120
Median	0.290	0.297	0.242	0.202	0.113	0.119	0.120
Min.	0.100	0.100	0.100	0.100	0.100	0.100	0.100
Max.	1.396	0.971	0.766	0.475	0.278	0.139	0.140
SD	0.338	0.258	0.189	0.123	0.067	0.027	0.030
**Pb [mg/kg d.m.]**							
Mean	72.758	125.632	75.717	60.082	60.844	19.320	12.200
Median	61.766	63.172	60.675	47.845	37.643	19.320	12.200
Min.	26.835	25.455	37.649	23.166	17.430	16.667	10.900
Max.	189.244	838.454	163.269	136.563	132.669	21.974	13.500
SD	41.518	198.522	41.537	37.482	47.953	3.753	1.290
**Hg [mg/kg d.m.]**							
Mean	0.185	0.134	0.102	0.318	0.109	0.273	0.090
Median	0.182	0.150	0.034	0.389	0.130	0.268	0.100
Min.	0.249	0.190	0.067	0.661	0.109	0.302	0.180
Max.	0.197	0.159	0.068	0.391	0.081	0.306	0.120
SD	0.032	0.032	0.031	0.034	0.031	0.034	0.030
**Cr [mg/kg d.m.]**							
Mean	12.843	12.796	12.939	13.407	13.378	19.301	19.560
Median	12.731	12.495	13.314	14.507	11.776	19.301	19.560
Min.	6.788	7.919	7.857	7.857	8.269	19.157	19.080
Max.	17.460	17.036	19.462	18.500	21.315	19.444	20.040
SD	2.951	2.766	3.480	4.387	4.732	0.203	0.680
**Ni [mg/kg d.m.]**							
Mean	8.018	8.142	8.321	8.720	9.500	14.099	14.950
Median	8.141	8.821	8.511	8.714	9.078	14.099	14.950
Min.	4.883	2.559	4.200	4.921	5.288	13.492	13.490
Max.	11.516	12.795	11.255	12.941	14.143	14.706	16.410
SD	2.054	2.546	1.895	2.050	2.813	0.607	1.460

			**Co [mg/kg d.m.]**				
Mean	3.898	3.921	4.170	4.252	4.417	5.040	5.300
Median	3.884	4.219	4.223	4.395	4.000	5.040	5.300
Min.	2.507	2.362	3.526	2.527	2.655	4.777	5.180
Max.	5.164	5.336	5.295	5.906	6.487	5.304	5.410
SD	0.828	0.928	0.471	1.068	1.340	0.264	0.120

			**Mn[mg/kg d.m.]**				
Mean	254.204	244.170	239.315	217.856	210.633	300.485	249.480
Median	254.974	242.794	246.985	235.554	214.286	300.485	249.480
Min.	175.952	143.031	143.520	123.425	125.769	292.381	230.360
Max.	331.793	354.213	312.891	292.471	275.654	308.588	268.590
SD	51.050	69.559	54.438	54.067	55.630	11.460	27.040
			**Zn [mg/kg d.m.]**				
Mean	147.334	147.016	140.999	102.842	87.265	64.471	48.670
Median	121.214	141.926	123.011	76.147	72.416	64.471	48.670
Min.	69.066	89.272	88.845	51.758	54.537	60.588	48.120
Max.	274.605	209.109	249.519	207.776	176.383	68.353	49.220
SD	64.613	36.255	44.235	52.637	43.124	3.882	0.550
			**Cu [mg/kg d.m.]**				
Mean	23.683	24.457	23.322	19.213	20.721	13.355	14.020
Median	19.563	20.364	22.311	18.884	18.075	13.355	14.020
Min.	11.380	6.594	15.079	13.400	10.769	12.103	12.80
Max.	60.078	54.762	36.273	31.297	37.450	14.606	15.230
SD	13.197	13.089	6.759	4.451	8.866	1.251	1.220
			**Fe [mg/kg d.m.]**				
Mean	9297.018	9039.099	9662.139	9691.489	10313.838	16501.389	16969.090
Median	8947.035	9237.464	9645.003	9405.735	9171.947	16501.389	16969.090
Min.	5396.887	4962.598	6777.778	5857.422	5505.906	16266.667	16894.530
Max.	13837.209	13185.484	12788.845	15490.000	17858.566	16736.111	17043.650
SD	2195.447	2188.504	1749.352	2999.189	4061.445	234.722	74.560
			**F [mg/kg d.m.]**				
Mean	88.927	73.323	16.163	16.423	12.757	12.738	12.721
Median	88.890	72.333	16.086	16.492	13.050	12.401	12.635
Min.	78.434	70.103	15.849	15.700	11.019	12.070	12.550
Max.	99.489	78.516	16.655	16.998	13.866	12.732	12.721
SD	22.437	9.846	2.293	2.066	1.576	0.331	0.085

Table S2. Statistical significance of the tested elements in the soil depending on the site of sampling. The obtained results were analyzed using the Statistica 10.0 software package. Wilcoxon tests were used to determine the significance of differences between the sampling sites and depths of measurement. A probability with $p \leq 0.05$ was considered statistically significant.

Depth (cm)	*statistical significance*
**Cd**	
**I vs III**	
0-20	p=0.04
60-80	p=0.04
**I vs IV**	
60-80	p=0.04
**II vs IV**	
80-100	p=0.04
**III vs IV**	
80-100	p=0.04
**Pb**	
**I vs II**	
60-80	p=0.03
80-100	p=0.03
**I vs III**	
40-60	p=0.004
60-80	p=0.004
80-100	p=0.03
**I vs IV**	
40-60	p=0.04
**II vs III**	
60-80	p=0.05
80-100	p=0.03
**II vs IV**	
60-80	p=0.004
80-100	p=0.004
**III vs IV**	
40-60	p=0.002
80-100	p=0.004
**Hg**	
**I vs II**	
40-60	p=0.004
60-80	p=0.03
80-100	p=0.03
**I vs III**	
40-60	p=0.004
60-80	p=0.004
80-100	P=0.03
**I vs IV**	
20-40	p=0.0002
40-60	p=0.004
60-80	p=0.004
80-100	p=0.004

II vs III	
0-20c	p=0.0002
20-40	p=0.0002
40-60	p=0.0002
60-80	p=0.004
**III vs IV**	
0-20	p=0.0002
20-40	p=0.0002
60-80	p=0.0002
**Cr**	
**I vs II**	
0-20	p=0.04
20-40	p=0.01
40-60	p=0.004
60-80	p=0.03
80-100	p=0.03
**I vs III**	
0-20	p=0.001
20-40	p=0.04
40-60	p=0.004
60-80	p=0.004
80-100	p=0.03
**I vs IV**	
0-20	p=0.01
40-60	p=0.004
60-80	p=0.04
**II vs III**	
0-20	p=0.01
**II vs IV**	
0-20	p=0.0002
20-40	p=0.002
40-60	p=0.0002
60-80	p=0.004
80-100	p=0.004
**III vs IV**	
0-20	p=0.0002
20-40	p=0.0002
40-60	p=0.0002
60-80	p=0.0002
80-100	p=0.004
**Ni**	
**I vs II**	
20-40	p=0.04
40-60	p=0.004
60-80	p=0.03
80-100	p=0.03
**I vs III**	
60-80	p=0.004
80-100	p=0.03
**I vs IV**	
20-40	p=0.04
**II vs III**	

80-100	p=0.03
**II vs IV**	
0-20	p=0.0001
20-40	p=0.002
40-60	p=0.01
60-80	p=0.05
80-100	p=0.004
**III vs IV**	
0-20	p=0.0001
20-40	p=0.01
60-80	p=0.002
80-100	p=0.004
**Fe**	
**I vs II**	
20-40	p=0.04
40-60	p=0.004
60-80	p=0.03
80-100	p=0.03
**I vs III**	
60-80	p=0.004
80-100	p=0.03
**I vs IV**	
40-60	p=0.05
60-80	p=0.05
**II vs III**	
0-20	p=0.01
**II vs IV**	
20-40	p=0.04
40-60	p=0.0002
60-80	p=0.004
80-100	p=0.004
**III vs IV**	
0-20	p=0.01
20-40	p=0.04
40-60	p=0.01
60-80	p=0.002
80-100	p=0.004
**Mn**	
**I vs II**	
40-60	p=0.004
60-80	p=0.03
80-100	p=0.03
**I vs III**	
0-20	p=0.01
20-40	p=0.0002
40-60	p=0.004
60-80	p=0.004
80-100	p=0.03
**I vs IV**	
40-60	p=0.05
80-100	p=0.05
**II vs IV**	

40-60	p=0.0002
60-80	p=0.05
80-100	p=0.004
**III vs IV**	
0-20	p=0.0002
40-60	p=0.0002
60-80	p=0.002
80-100	p=0.004
**Zn**	
**I vs II**	
80-100	p=0.03
**I vs III**	
80-100	p=0.03
**I vs IV**	
60-80	p=0.004
**II vs III**	
0-20	p=0.0002
60-80	p=0.004
80-100	p=0.03
**II vs IV**	
60-80cm	p=0.004
80-100	p=0.004
**III vs IV**	
0-20	p=0.01
20-40	p=0.01
80-100	p=0.004
**Cu**	
**I vs II**	
40-60	p=0.05
80-100	p=0.03
**I vs III**	
40-60	p=0.004
80-100	p=0.03
**I vs IV**	
40-60	p=0.004
80-100	p=0.004
**II vs III**	
20-40	p=0.0002
40-60	p=0.0002
80-100	p=0.03
**II vs IV**	
0-20	p=0.002
20-40	p=0.0002
40-60	p=0.0002
**III vs IV**	
0-20	p=0.002
**Co**	
**I vs II**	
60-80	p=0.03
80-100	p=0.03
**I vs III**	

60-80	p=0.004
80-100	p=0.03
**I vs IV**	
40-60	p=0.05
60-80	p=0.004
**II vs IV**	
0-20	p=0.04
20-40	p=0.01
40-60	p=0.01
60-80	p=0.004
80-100	p=0.004
**III vs IV**	
0-20	p=0.04
40-60	p=0.01
60-80	p=0.002

Table S3. Statistical significance of the tested elements in the soil depending on the depth of sampling. The obtained results were analyzed using the Statistica 10.0 software package. Mann-Whitney U tests were used to determine the significance of differences between the sampling sites and depths of measurement. A probability with $p \leq 0.05$ was considered statistically significant.

Depth **(cm)**	*statistical significance*
**Cd**	
**III**	
0-20 vs60-80	p=0.01
20-40 vs 40-60	p=0.03
20-40 vs 60-80	p=0.01
**IV**	
0-20 vs 80-100	p=0.04
20-40 vs 40-60	p=0.01
20-40 vs 60-80	p=0.01
**Pb**	
**II**	
0-20 vs 20-40	p=0.04
20-40 vs 60-80	p=0.01
20-40 vs 80-100	p=0.01
60-80 vs 80-100	p=0.04
**III**	
0-20 vs 40-60	p=0.01
0-20 vs 60-80	p=0.01
20-40 vs 40-60	p=0.01
20-40 vs 60-80	p=0.01
40-60 vs 60-80	p=0.04
**IV**	
0-20 vs 20-40	p=0.04
20-40 vs 60-80	p=0.01
20-40 vs 80-100	p=0.01
60-80 vs 80-100	p=0.04
**Hg**	
**IV**	
0-20 vs 20-40	p=0.04

20-40 vs 60-80	p=0.01
20-40 vs 80-100	p=0.01
**Cr**	
**I**	
0-20 vs 20-40	p=0.04
**II**	
0-20 vs 40-60	p=0.04
**III**	
0-20 vs 20-40	p=0.04
0-20 vs 60-80	p=0.04
20-40 vs 60-80	p=0.01
40-60 vs 60-80	p=0.04
**IV**	
0-20vs 20-40	p=0.01
0-20 vs 60-80	p=0.04
**Ni**	
**III**	
20-40 vs 60-80	p=0.01
40-60 vs 60-80	p=0.01
**IV**	
0-20 vs 60-80	p=0.01
0-20 vs 80-100	p=0.01
20-40 vs 60-80	p=0.01
20-40 vs 80-100	p=0.04
**Fe**	
**II**	
0-20 vs 40-60	p=0.04
**III**	
0-20 vs 60-80	p=0.01
20-40 vs 60-80	p=0.01
40-60 vs 60-80	p=0.01
**IV**	
40-60 vs 80-100	p=0.04
60-80 vs 80-100	p=0.01
**Zn**	
**II**	
0-20 vs 20-40	p=0.01
**III**	
0-20 vs 40-60	p=0.01
0-20 vs 60-80	p=0.01
20-40 vs 40-60	p=0.01
20-40 vs 60-80	p=0.01
40-60 vs 60-80	p=0.04
**IV**	
60-80 vs 80-100	p=0.04
**Cu**	
**II**	
0-20 vs 20-40	p=0.01
0-20 vs 40-60	p=0.01
**III**	

0-20 vs 20-40	p=0.04
0-20 vs 40-60	p=0.01
0-20 vs 60-80	p=0.01
20-40 vs 40-60	p=0.01
**IV**	
0-20 vs 60-80	p=0.04
20-40 vs 60-80	p=0.01
20-40 vs 80-100	p=0.01
**Co**	
**III**	
0-20 vs 60-80	p=0.01
20-40 vs 60-80	p=0.01
40-60 vs 60-80	p=0.01
**IV**	
20-40 vs 60-80	p=0.01
60-80 vs 80-100	p=0.01
**Mn**	
**I**	
0-20 vs 20-40	p=0.01
**III**	
0-20 vs 40-60	p=0.04
0-20 vs 60-80	p=0.04
20-40 vs 60-80	p=0.01
**IV**	
0-20 vs 40-60	p=0.04
20-40 vs 60-80	p=0.04
20-40 vs 80-100	p=0.02
40-60 vs 80-100	p=0.04
60-80 vs 80-100	p=0.04
**F**	
**I**	
0-20 vs 40-60	p=0.0001
0-20 vs 60-80	p=0.0001
0-20 vs 80-100	p=0.0002
0-20 vs 100-120	p=0.00025
20-40 vs 40-60	p=0.001
20-40 vs 60-80	p=0.0022
20-40 vs 80-100	p=0.00041
20-40 vs 100-120	p=0.00041
**II**	
0-20 vs 40-60	p=0.0001
0-20 vs 60-80	p=0.0001
0-20 vs 80-100	p=0.0002
0-20 vs 100-120	p=0.00025
20-40 vs 40-60	p=0.001
20-40 vs 60-80	p=0.0022
20-40 vs 80-100	p=0.00041
20-40 vs 100-120	p=0.00041
**III**	
0-20 vs 40-60	p=0.0001
0-20 vs 60-80	p=0.0001

0-20 vs 80-100	p=0.0002
0-20 vs 100-120	p=0.00025
20-40 vs 40-60	p=0.001
20-40 vs 60-80	p=0.0022
20-40 vs 80-100	p=0.00041
20-40 vs 100-120	p=0.00041
**IV**	
0-20 vs 40-60	p=0.0001
0-20 vs 60-80	p=0.0001
0-20 vs 80-100	p=0.0002
0-20 vs 100-120	p=0.00025
20-40 vs 40-60	p=0.001
20-40 vs 60-80	p=0.0022
20-40 vs 80-100	p=0.00041
20-40 vs 100-120	p=0.00041
